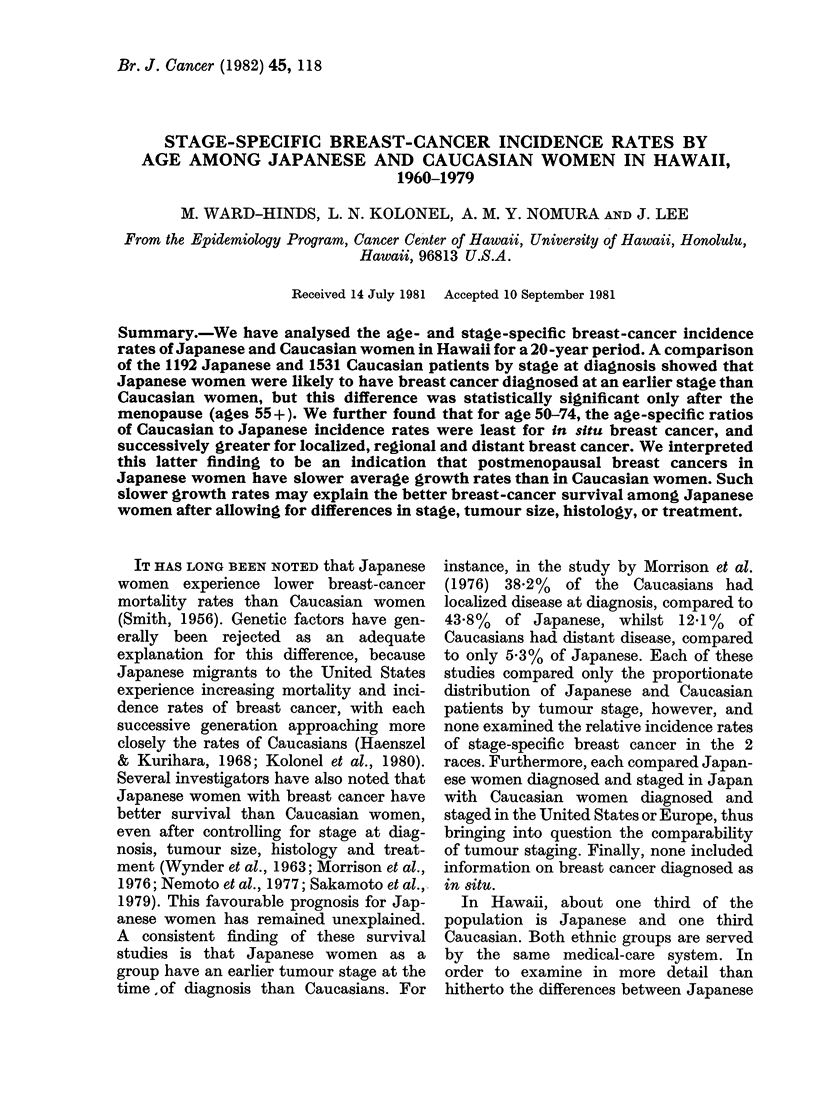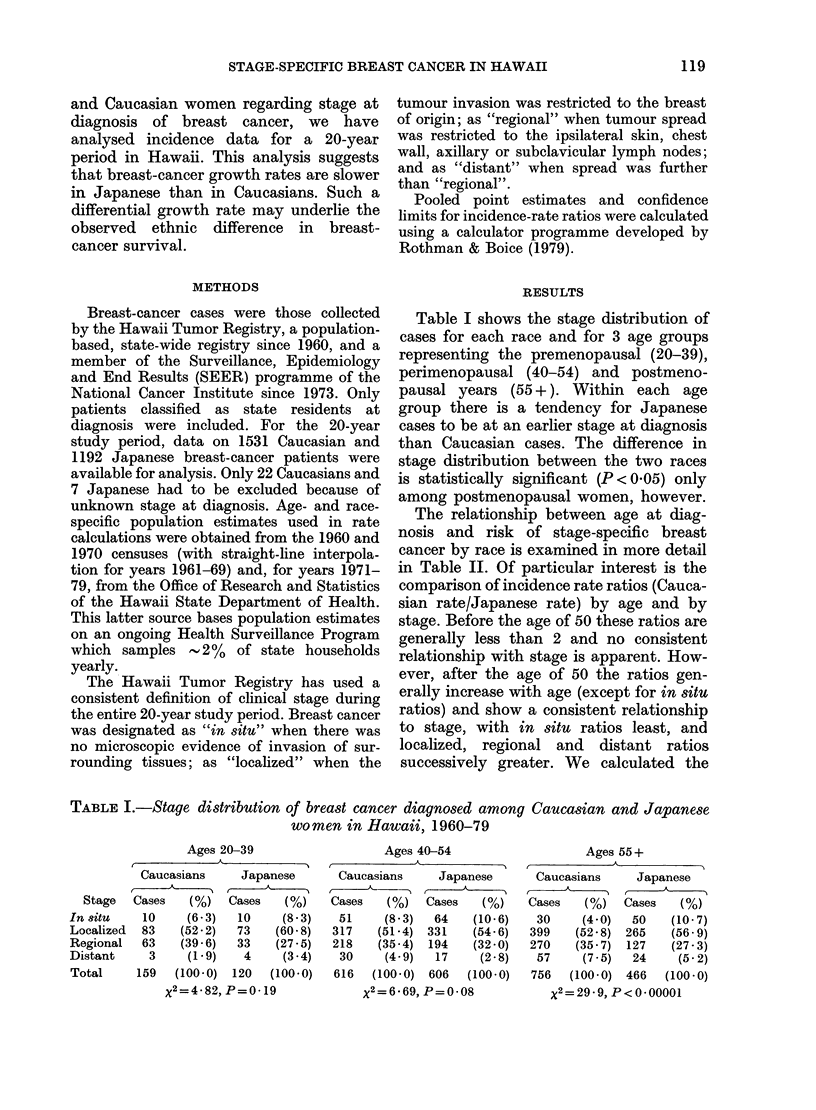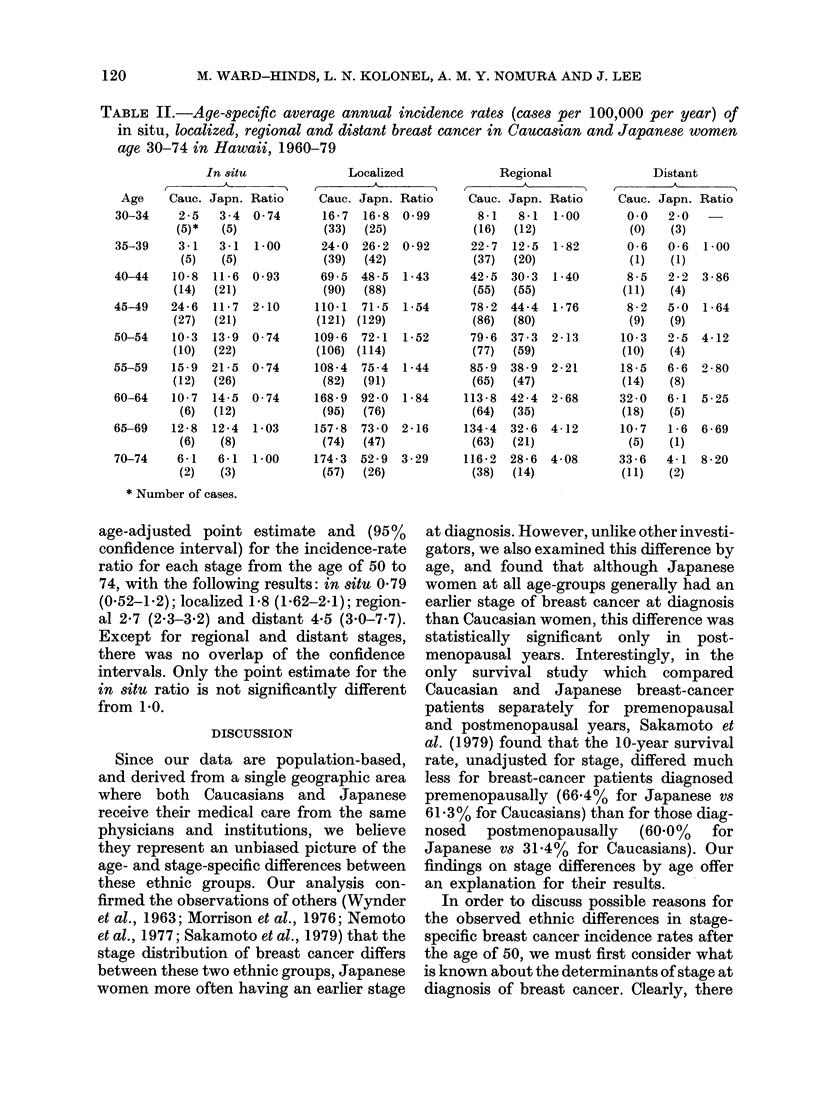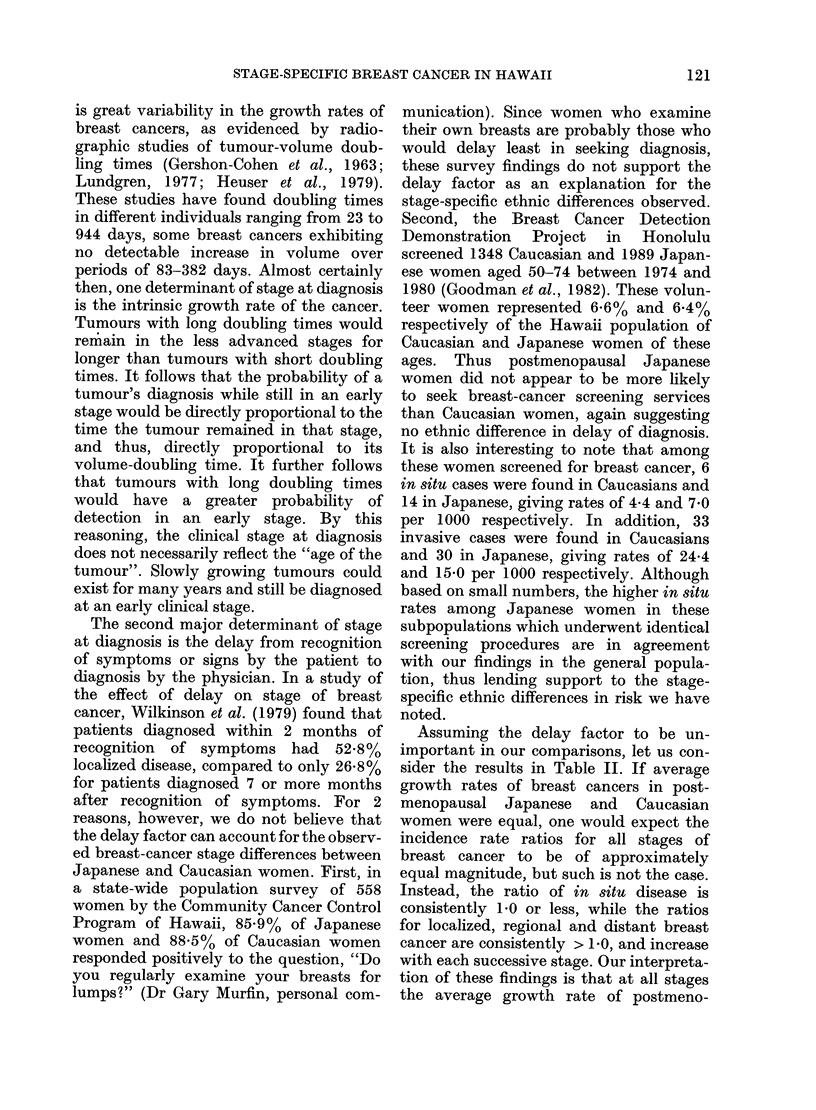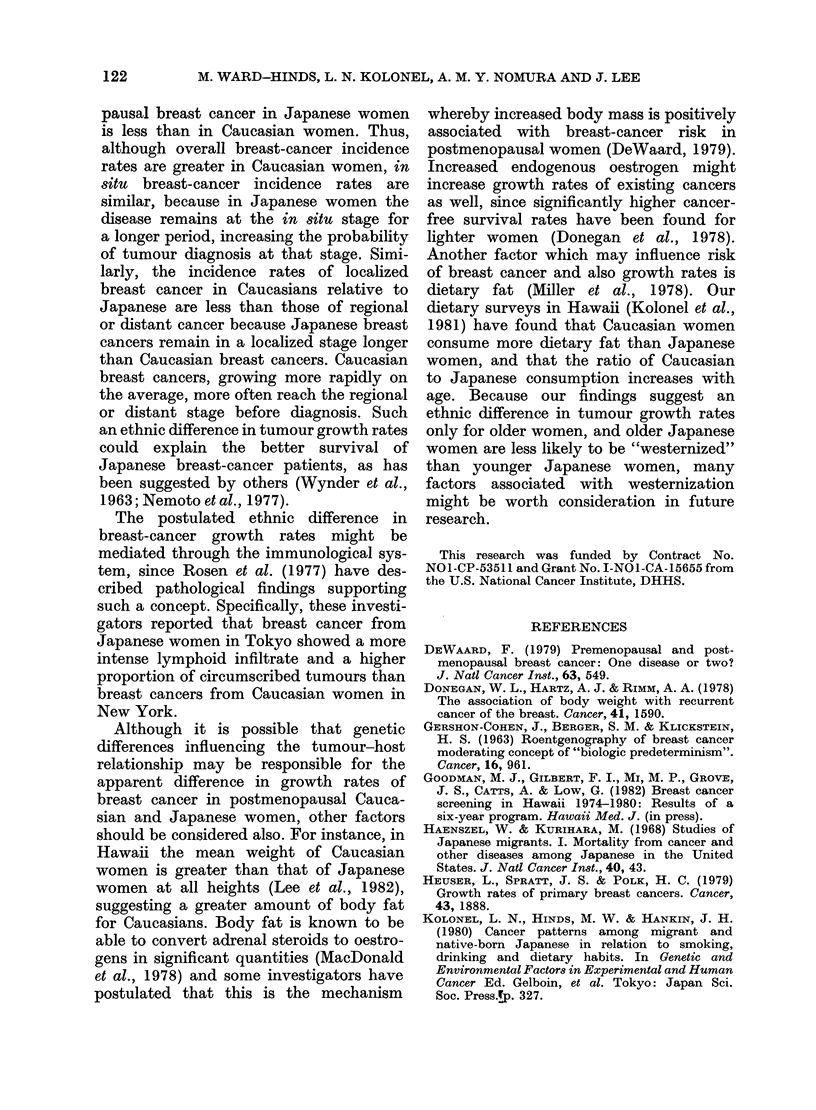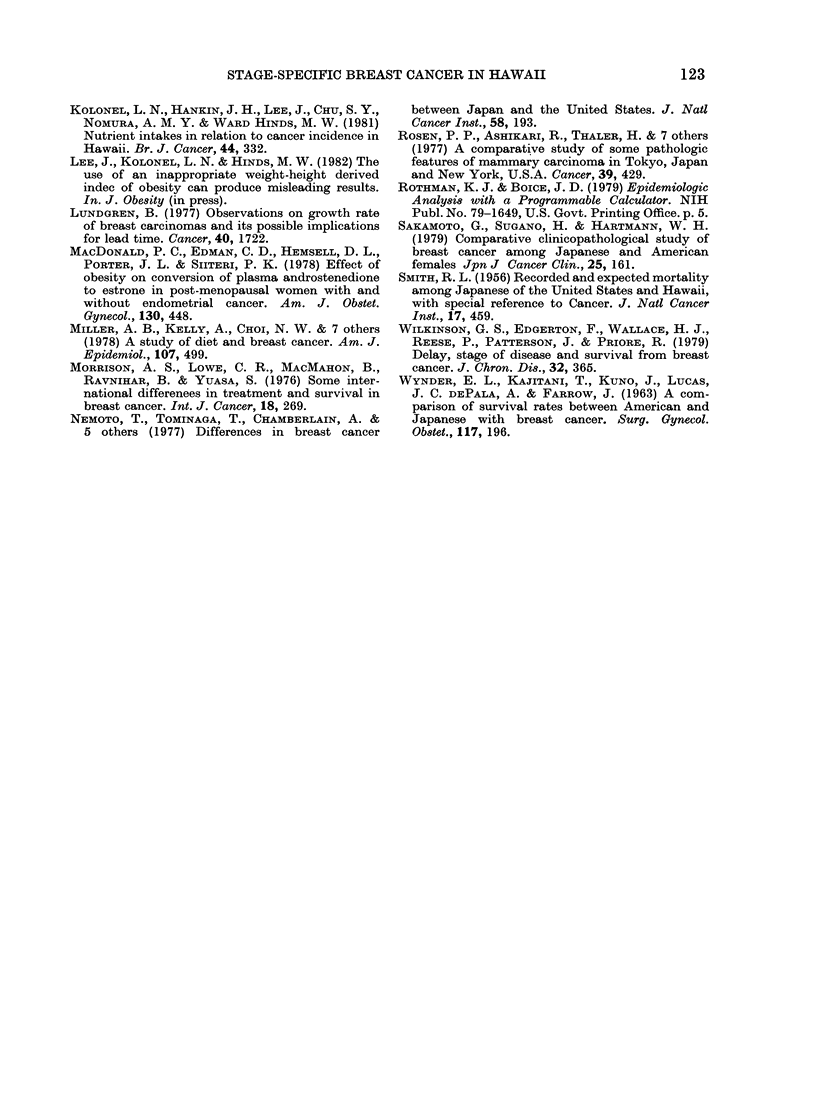# Stage-specific breast-cancer incidence rates by age among Japanese and Caucasian women in Hawaii, 1960-1979.

**DOI:** 10.1038/bjc.1982.14

**Published:** 1982-01

**Authors:** M. Ward-Hinds, L. N. Kolonel, A. M. Nomura, J. Lee

## Abstract

We have analysed the age- and stage-specific breast-cancer incidence rates of Japanese and Caucasian women in Hawaii for a 20-year period. A comparison of the 1192 Japanese and 1531 Caucasian patients by stage at diagnosis showed that Japanese women were likely to have breast cancer diagnosed at an earlier stage than Caucasian women, but this difference was statistically significant only after the menopause (ages 55+). We further found that for age 50-74, the age-specific ratios of Caucasian to Japanese incidence rates were least for in situ breast cancer, and successively greater for localized, regional and distant breast cancer. We interpreted this latter finding to be an indication that postmenopausal breast cancers in Japanese women have slower average growth rates than in Caucasian women. Such slower growth rates may explain the better breast-cancer survival among Japanese women after allowing for differences in stage, tumour size, histology, or treatment.


*Br. J. Cancer* (1982) **45**, 118

### STAGE-SPECIFIC BREAST-CANCER INCIDENCE RATES BY AGE AMONG JAPANESE AND CAUCASIAN WOMEN IN HAWAII, 1960–1979

M. WARD-HINDS, L. N. KOLONEL, A. M. Y. NOMURA AND J. LEE

*From the Epidemiology Program, Cancer Center of Hawaii, University of Hawaii, Honolulu, Hawaii,* 96813 *U.S.A.*

Received 14 July 1981 Accepted 10 September 1981

**Summary.—We have analysed the age- and stage-specific breast-cancer incidence rates of Japanese and Caucasian women in Hawaii for a 20-year period. A comparison of the 1192 Japanese and 1531 Caucasian patients by stage at diagnosis showed that Japanese women were likely to have breast cancer diagnosed at an earlier stage than Caucasian women, but this difference was statistically significant only after the menopause (ages 55+). We further found that for age 50–74, the age-specific ratios of Caucasian to Japanese incidence rates were least for *in situ* breast cancer, and successively greater for localized, regional and distant breast cancer. We interpreted this latter finding to be an indication that postmenopausal breast cancers in Japanese women have slower average growth rates than in Caucasian women. Such slower growth rates may explain the better breast-cancer survival among Japanese women after allowing for differences in stage, tumour size, histology, or treatment.**

IT HAS LONG BEEN NOTED that Japanese women experience lower breast-cancer mortality rates than Caucasian women (Smith, 1956). Genetic factors have generally been rejected as an adequate explanation for this difference, because Japanese migrants to the United States experience increasing mortality and incidence rates of breast cancer, with each successive generation approaching more closely the rates of Caucasians (Haenszel & Kurihara, 1968; Kolonel *et al.*, 1980). Several investigators have also noted that Japanese women with breast cancer have better survival than Caucasian women, even after controlling for stage at diagnosis, tumour size, histology and treatment (Wynder *et al.*, 1963; Morrison *et al.*, 1976; Nemoto *et al.*, 1977; Sakamoto *et al.*, 1979). This favourable prognosis for Japanese women has remained unexplained. A consistent finding of these survival studies is that Japanese women as a group have an earlier tumour stage at the time of diagnosis than Caucasians. For instance, in the study by Morrison *et al.* (1976) 38·2% of the Caucasians had localized disease at diagnosis, compared to 43·8% of Japanese, whilst 12·1% of Caucasians had distant disease, compared to only 5·3% of Japanese. Each of these studies compared only the proportionate distribution of Japanese and Caucasian patients by tumour stage, however, and none examined the relative incidence rates of stage-specific breast cancer in the 2 races. Furthermore, each compared Japanese women diagnosed and staged in Japan with Caucasian women diagnosed and staged in the United States or Europe, thus bringing into question the comparability of tumour staging. Finally, none included information on breast cancer diagnosed as *in situ*.

In Hawaii, about one third of the population is Japanese and one third Caucasian. Both ethnic groups are served by the same medical-care system. In order to examine in more detail than hitherto the differences between Japanese and Caucasian women regarding stage at diagnosis of breast cancer, we have analysed incidence data for a 20-year period in Hawaii. This analysis suggests that breast-cancer growth rates are slower in Japanese than in Caucasians. Such a differential growth rate may underlie the observed ethnic difference in breast-cancer survival.

### METHODS

Breast-cancer cases were those collected by the Hawaii Tumor Registry, a population-based, state-wide registry since 1960, and a member of the Surveillance, Epidemiology and End Results (SEER) programme of the National Cancer Institute since 1973. Only patients classified as state residents at diagnosis were included. For the 20-year study period, data on 1531 Caucasian and 1192 Japanese breast-cancer patients were available for analysis. Only 22 Caucasians and 7 Japanese had to be excluded because of unknown stage at diagnosis. Age- and race-specific population estimates used in rate calculations were obtained from the 1960 and 1970 censuses (with straight-line interpolation for years 1961–69) and, for years 1971–79, from the Office of Research and Statistics of the Hawaii State Department of Health. This latter source bases population estimates on an ongoing Health Surveillance Program which samples ~2% of state households yearly.

The Hawaii Tumor Registry has used a consistent definition of clinical stage during the entire 20-year study period. Breast cancer was designated as "*in situ*" when there was no microscopic evidence of invasion of surrounding tissues; as "localized" when the tumour invasion was restricted to the breast of origin; as "regional" when tumour spread was restricted to the ipsilateral skin, chest wall, axillary or subclavicular lymph nodes; and as "distant" when spread was further than "regional".

Pooled point estimates and confidence limits for incidence-rate ratios were calculated using a calculator programme developed by Rothman & Boice (1979).

### RESULTS

Table I shows the stage distribution of cases for each race and for 3 age groups representing the premenopausal (20–39), perimenopausal (40–54) and postmenopausal years (55+). Within each age group there is a tendency for Japanese cases to be at an earlier stage at diagnosis than Caucasian cases. The difference in stage distribution between the two races is statistically significant ($P < 0{\cdot}05$) only among postmenopausal women, however.

The relationship between age at diagnosis and risk of stage-specific breast cancer by race is examined in more detail in Table II. Of particular interest is the comparison of incidence rate ratios (Caucasian rate/Japanese rate) by age and by stage. Before the age of 50 these ratios are generally less than 2 and no consistent relationship with stage is apparent. However, after the age of 50 the ratios generally increase with age (except for *in situ* ratios) and show a consistent relationship to stage, with *in situ* ratios least, and localized, regional and distant ratios successively greater. We calculated the

TABLE I.—*Stage distribution of breast cancer diagnosed among Caucasian and Japanese women in Hawaii*, 1960–79

| | Ages 20–39 | | | | Ages 40–54 | | | | Ages 55+ | | | |
|---|---|---|---|---|---|---|---|---|---|---|---|---|
| | Caucasians | | Japanese | | Caucasians | | Japanese | | Caucasians | | Japanese | |
| Stage | Cases | (%) | Cases | (%) | Cases | (%) | Cases | (%) | Cases | (%) | Cases | (%) |
| *In situ* | 10 | (6·3) | 10 | (8·3) | 51 | (8·3) | 64 | (10·6) | 30 | (4·0) | 50 | (10·7) |
| Localized | 83 | (52·2) | 73 | (60·8) | 317 | (51·4) | 331 | (54·6) | 399 | (52·8) | 265 | (56·9) |
| Regional | 63 | (39·6) | 33 | (27·5) | 218 | (35·4) | 194 | (32·0) | 270 | (35·7) | 127 | (27·3) |
| Distant | 3 | (1·9) | 4 | (3·4) | 30 | (4·9) | 17 | (2·8) | 57 | (7·5) | 24 | (5·2) |
| Total | 159 | (100·0) | 120 | (100·0) | 616 | (100·0) | 606 | (100·0) | 756 | (100·0) | 466 | (100·0) |
| | $\chi^2 = 4{\cdot}82$, $P = 0{\cdot}19$ | | | | $\chi^2 = 6{\cdot}69$, $P = 0{\cdot}08$ | | | | $\chi^2 = 29{\cdot}9$, $P < 0{\cdot}00001$ | | | |

TABLE II.—*Age-specific average annual incidence rates (cases per* 100,000 *per year) of* in situ, *localized, regional and distant breast cancer in Caucasian and Japanese women age* 30–74 *in Hawaii,* 1960–79

| | *In situ* | | | Localized | | | Regional | | | Distant | | |
|---|---|---|---|---|---|---|---|---|---|---|---|---|
| Age | Cauc. | Japn. | Ratio | Cauc. | Japn. | Ratio | Cauc. | Japn. | Ratio | Cauc. | Japn. | Ratio |
| 30–34 | 2·5 | 3·4 | 0·74 | 16·7 | 16·8 | 0·99 | 8·1 | 8·1 | 1·00 | 0·0 | 2·0 | — |
| | (5)* | (5) | | (33) | (25) | | (16) | (12) | | (0) | (3) | |
| 35–39 | 3·1 | 3·1 | 1·00 | 24·0 | 26·2 | 0·92 | 22·7 | 12·5 | 1·82 | 0·6 | 0·6 | 1·00 |
| | (5) | (5) | | (39) | (42) | | (37) | (20) | | (1) | (1) | |
| 40–44 | 10·8 | 11·6 | 0·93 | 69·5 | 48·5 | 1·43 | 42·5 | 30·3 | 1·40 | 8·5 | 2·2 | 3·86 |
| | (14) | (21) | | (90) | (88) | | (55) | (55) | | (11) | (4) | |
| 45–49 | 24·6 | 11·7 | 2·10 | 110·1 | 71·5 | 1·54 | 78·2 | 44·4 | 1·76 | 8·2 | 5·0 | 1·64 |
| | (27) | (21) | | (121) | (129) | | (86) | (80) | | (9) | (9) | |
| 50–54 | 10·3 | 13·9 | 0·74 | 109·6 | 72·1 | 1·52 | 79·6 | 37·3 | 2·13 | 10·3 | 2·5 | 4·12 |
| | (10) | (22) | | (106) | (114) | | (77) | (59) | | (10) | (4) | |
| 55–59 | 15·9 | 21·5 | 0·74 | 108·4 | 75·4 | 1·44 | 85·9 | 38·9 | 2·21 | 18·5 | 6·6 | 2·80 |
| | (12) | (26) | | (82) | (91) | | (65) | (47) | | (14) | (8) | |
| 60–64 | 10·7 | 14·5 | 0·74 | 168·9 | 92·0 | 1·84 | 113·8 | 42·4 | 2·68 | 32·0 | 6·1 | 5·25 |
| | (6) | (12) | | (95) | (76) | | (64) | (35) | | (18) | (5) | |
| 65–69 | 12·8 | 12·4 | 1·03 | 157·8 | 73·0 | 2·16 | 134·4 | 32·6 | 4·12 | 10·7 | 1·6 | 6·69 |
| | (6) | (8) | | (74) | (47) | | (63) | (21) | | (5) | (1) | |
| 70–74 | 6·1 | 6·1 | 1·00 | 174·3 | 52·9 | 3·29 | 116·2 | 28·6 | 4·08 | 33·6 | 4·1 | 8·20 |
| | (2) | (3) | | (57) | (26) | | (38) | (14) | | (11) | (2) | |

* Number of cases.

age-adjusted point estimate and (95% confidence interval) for the incidence-rate ratio for each stage from the age of 50 to 74, with the following results: *in situ* 0·79 (0·52–1·2); localized 1·8 (1·62–2·1); regional 2·7 (2·3–3·2) and distant 4·5 (3·0–7·7). Except for regional and distant stages, there was no overlap of the confidence intervals. Only the point estimate for the *in situ* ratio is not significantly different from 1·0.

### DISCUSSION

Since our data are population-based, and derived from a single geographic area where both Caucasians and Japanese receive their medical care from the same physicians and institutions, we believe they represent an unbiased picture of the age- and stage-specific differences between these ethnic groups. Our analysis confirmed the observations of others (Wynder *et al.*, 1963; Morrison *et al.*, 1976; Nemoto *et al.*, 1977; Sakamoto *et al.*, 1979) that the stage distribution of breast cancer differs between these two ethnic groups, Japanese women more often having an earlier stage at diagnosis. However, unlike other investigators, we also examined this difference by age, and found that although Japanese women at all age-groups generally had an earlier stage of breast cancer at diagnosis than Caucasian women, this difference was statistically significant only in postmenopausal years. Interestingly, in the only survival study which compared Caucasian and Japanese breast-cancer patients separately for premenopausal and postmenopausal years, Sakamoto *et al.* (1979) found that the 10-year survival rate, unadjusted for stage, differed much less for breast-cancer patients diagnosed premenopausally (66·4% for Japanese *vs* 61·3% for Caucasians) than for those diagnosed postmenopausally (60·0% for Japanese *vs* 31·4% for Caucasians). Our findings on stage differences by age offer an explanation for their results.

In order to discuss possible reasons for the observed ethnic differences in stage-specific breast cancer incidence rates after the age of 50, we must first consider what is known about the determinants of stage at diagnosis of breast cancer. Clearly, there is great variability in the growth rates of breast cancers, as evidenced by radiographic studies of tumour-volume doubling times (Gershon-Cohen *et al.*, 1963; Lundgren, 1977; Heuser *et al.*, 1979). These studies have found doubling times in different individuals ranging from 23 to 944 days, some breast cancers exhibiting no detectable increase in volume over periods of 83–382 days. Almost certainly then, one determinant of stage at diagnosis is the intrinsic growth rate of the cancer. Tumours with long doubling times would remain in the less advanced stages for longer than tumours with short doubling times. It follows that the probability of a tumour's diagnosis while still in an early stage would be directly proportional to the time the tumour remained in that stage, and thus, directly proportional to its volume-doubling time. It further follows that tumours with long doubling times would have a greater probability of detection in an early stage. By this reasoning, the clinical stage at diagnosis does not necessarily reflect the "age of the tumour". Slowly growing tumours could exist for many years and still be diagnosed at an early clinical stage.

The second major determinant of stage at diagnosis is the delay from recognition of symptoms or signs by the patient to diagnosis by the physician. In a study of the effect of delay on stage of breast cancer, Wilkinson *et al.* (1979) found that patients diagnosed within 2 months of recognition of symptoms had 52·8% localized disease, compared to only 26·8% for patients diagnosed 7 or more months after recognition of symptoms. For 2 reasons, however, we do not believe that the delay factor can account for the observed breast-cancer stage differences between Japanese and Caucasian women. First, in a state-wide population survey of 558 women by the Community Cancer Control Program of Hawaii, 85·9% of Japanese women and 88·5% of Caucasian women responded positively to the question, "Do you regularly examine your breasts for lumps?" (Dr Gary Murfin, personal communication). Since women who examine their own breasts are probably those who would delay least in seeking diagnosis, these survey findings do not support the delay factor as an explanation for the stage-specific ethnic differences observed. Second, the Breast Cancer Detection Demonstration Project in Honolulu screened 1348 Caucasian and 1989 Japanese women aged 50–74 between 1974 and 1980 (Goodman *et al.*, 1982). These volunteer women represented 6·6% and 6·4% respectively of the Hawaii population of Caucasian and Japanese women of these ages. Thus postmenopausal Japanese women did not appear to be more likely to seek breast-cancer screening services than Caucasian women, again suggesting no ethnic difference in delay of diagnosis. It is also interesting to note that among these women screened for breast cancer, 6 *in situ* cases were found in Caucasians and 14 in Japanese, giving rates of 4·4 and 7·0 per 1000 respectively. In addition, 33 invasive cases were found in Caucasians and 30 in Japanese, giving rates of 24·4 and 15·0 per 1000 respectively. Although based on small numbers, the higher *in situ* rates among Japanese women in these subpopulations which underwent identical screening procedures are in agreement with our findings in the general population, thus lending support to the stage-specific ethnic differences in risk we have noted.

Assuming the delay factor to be unimportant in our comparisons, let us consider the results in Table II. If average growth rates of breast cancers in postmenopausal Japanese and Caucasian women were equal, one would expect the incidence rate ratios for all stages of breast cancer to be of approximately equal magnitude, but such is not the case. Instead, the ratio of *in situ* disease is consistently 1·0 or less, while the ratios for localized, regional and distant breast cancer are consistently $> 1{\cdot}0$, and increase with each successive stage. Our interpretation of these findings is that at all stages the average growth rate of postmenopausal breast cancer in Japanese women is less than in Caucasian women. Thus, although overall breast-cancer incidence rates are greater in Caucasian women, *in situ* breast-cancer incidence rates are similar, because in Japanese women the disease remains at the *in situ* stage for a longer period, increasing the probability of tumour diagnosis at that stage. Similarly, the incidence rates of localized breast cancer in Caucasians relative to Japanese are less than those of regional or distant cancer because Japanese breast cancers remain in a localized stage longer than Caucasian breast cancers. Caucasian breast cancers, growing more rapidly on the average, more often reach the regional or distant stage before diagnosis. Such an ethnic difference in tumour growth rates could explain the better survival of Japanese breast-cancer patients, as has been suggested by others (Wynder *et al.*, 1963; Nemoto *et al.*, 1977).

The postulated ethnic difference in breast-cancer growth rates might be mediated through the immunological system, since Rosen *et al.* (1977) have described pathological findings supporting such a concept. Specifically, these investigators reported that breast cancer from Japanese women in Tokyo showed a more intense lymphoid infiltrate and a higher proportion of circumscribed tumours than breast cancers from Caucasian women in New York.

Although it is possible that genetic differences influencing the tumour–host relationship may be responsible for the apparent difference in growth rates of breast cancer in postmenopausal Caucasian and Japanese women, other factors should be considered also. For instance, in Hawaii the mean weight of Caucasian women is greater than that of Japanese women at all heights (Lee *et al.*, 1982), suggesting a greater amount of body fat for Caucasians. Body fat is known to be able to convert adrenal steroids to oestrogens in significant quantities (MacDonald *et al.*, 1978) and some investigators have postulated that this is the mechanism whereby increased body mass is positively associated with breast-cancer risk in postmenopausal women (DeWaard, 1979). Increased endogenous oestrogen might increase growth rates of existing cancers as well, since significantly higher cancer-free survival rates have been found for lighter women (Donegan *et al.*, 1978). Another factor which may influence risk of breast cancer and also growth rates is dietary fat (Miller *et al.*, 1978). Our dietary surveys in Hawaii (Kolonel *et al.*, 1981) have found that Caucasian women consume more dietary fat than Japanese women, and that the ratio of Caucasian to Japanese consumption increases with age. Because our findings suggest an ethnic difference in tumour growth rates only for older women, and older Japanese women are less likely to be "westernized" than younger Japanese women, many factors associated with westernization might be worth consideration in future research.

This research was funded by Contract No. NO1-CP-53511 and Grant No. I-NO1-CA-15655 from the U.S. National Cancer Institute, DHHS.

REFERENCES

DeWaard, F. (1979) Premenopausal and postmenopausal breast cancer: One disease or two? *J. Natl Cancer Inst.*, **63**, 549.

Donegan, W. L., Hartz, A. J. & Rimm, A. A. (1978) The association of body weight with recurrent cancer of the breast. *Cancer*, **41**, 1590.

Gershon-Cohen, J., Berger, S. M. & Klickstein, H. S. (1963) Roentgenography of breast cancer moderating concept of "biologic predeterminism". *Cancer*, **16**, 961.

Goodman, M. J., Gilbert, F. I., Mi, M. P., Grove, J. S., Catts, A. & Low, G. (1982) Breast cancer screening in Hawaii 1974–1980: Results of a six-year program. *Hawaii Med. J.* (in press).

Haenszel, W. & Kurihara, M. (1968) Studies of Japanese migrants. I. Mortality from cancer and other diseases among Japanese in the United States. *J. Natl Cancer Inst.*, **40**, 43.

Heuser, L., Spratt, J. S. & Polk, H. C. (1979) Growth rates of primary breast cancers. *Cancer*, **43**, 1888.

Kolonel, L. N., Hinds, M. W. & Hankin, J. H. (1980) Cancer patterns among migrant and native-born Japanese in relation to smoking, drinking and dietary habits. In *Genetic and Environmental Factors in Experimental and Human Cancer* Ed. Gelboin, *et al.* Tokyo: Japan Sci. Soc. Press. p. 327.

KOLONEL, L. N., HANKIN, J. H., LEE, J., CHU, S. Y., NOMURA, A. M. Y. & WARD HINDS, M. W. (1981) Nutrient intakes in relation to cancer incidence in Hawaii. *Br. J. Cancer*, **44**, 332.

LEE, J., KOLONEL, L. N. & HINDS, M. W. (1982) The use of an inappropriate weight-height derived indec of obesity can produce misleading results. *In. J. Obesity* (in press).

LUNDGREN, B. (1977) Observations on growth rate of breast carcinomas and its possible implications for lead time. *Cancer*, **40**, 1722.

MACDONALD, P. C., EDMAN, C. D., HEMSELL, D. L., PORTER, J. L. & SIITERI, P. K. (1978) Effect of obesity on conversion of plasma androstenedione to estrone in post-menopausal women with and without endometrial cancer. *Am. J. Obstet. Gynecol.*, **130**, 448.

MILLER, A. B., KELLY, A., CHOI, N. W. & 7 others (1978) A study of diet and breast cancer. *Am. J. Epidemiol.*, **107**, 499.

MORRISON, A. S., LOWE, C. R., MACMAHON, B., RAVNIHAR, B. & YUASA, S. (1976) Some international differenees in treatment and survival in breast cancer. *Int. J. Cancer*, **18**, 269.

NEMOTO, T., TOMINAGA, T., CHAMBERLAIN, A. & 5 others (1977) Differences in breast cancer between Japan and the United States. *J. Natl Cancer Inst.*, **58**, 193.

ROSEN, P. P., ASHIKARI, R., THALER, H. & 7 others (1977) A comparative study of some pathologic features of mammary carcinoma in Tokyo, Japan and New York, U.S.A. *Cancer*, **39**, 429.

ROTHMAN, K. J. & BOICE, J. D. (1979) *Epidemiologic Analysis with a Programmable Calculator.* NIH Publ. No. 79–1649, U.S. Govt. Printing Office. p. 5.

SAKAMOTO, G., SUGANO, H. & HARTMANN, W. H. (1979) Comparative clinicopathological study of breast cancer among Japanese and American females *Jpn J Cancer Clin.*, **25**, 161.

SMITH, R. L. (1956) Recorded and expected mortality among Japanese of the United States and Hawaii, with special reference to Cancer. *J. Natl Cancer Inst.*, **17**, 459.

WILKINSON, G. S., EDGERTON, F., WALLACE, H. J., REESE, P., PATTERSON, J. & PRIORE, R. (1979) Delay, stage of disease and survival from breast cancer. *J. Chron. Dis.*, **32**, 365.

WYNDER, E. L., KAJITANI, T., KUNO, J., LUCAS, J. C. DEPALA, A. & FARROW, J. (1963) A comparison of survival rates between American and Japanese with breast cancer. *Surg. Gynecol. Obstet.*, **117**, 196.